# Wfs1-deficient mice display altered function of serotonergic system and increased behavioral response to antidepressants

**DOI:** 10.3389/fnins.2013.00132

**Published:** 2013-07-31

**Authors:** Tanel Visnapuu, Sirli Raud, Maarja Loomets, Riin Reimets, Silva Sütt, Hendrik Luuk, Mario Plaas, Sulev Kõks, Vallo Volke, Aet Alttoa, Jaanus Harro, Eero Vasar

**Affiliations:** ^1^Department of Physiology, University of TartuTartu, Estonia; ^2^Centre for Excellence in Translational Medicine, University of TartuTartu, Estonia; ^3^Department of Psychology, Estonian Centre of Behavioral and Health Sciences, University of TartuTartu, Estonia

**Keywords:** behavioral despair, forced swimming test, tail suspension test, imipramine, paroxetine, serotonin reuptake transporter, noradrenaline reuptake transporter, Wfs1-deficient mice

## Abstract

It has been shown that mutations in the WFS1 gene make humans more susceptible to mood disorders. Besides that, mood disorders are associated with alterations in the activity of serotonergic and noradrenergic systems. Therefore, in this study, the effects of imipramine, an inhibitor of serotonin (5-HT) and noradrenaline (NA) reuptake, and paroxetine, a selective inhibitor of 5-HT reuptake, were studied in tests of behavioral despair. The tail suspension test (TST) and forced swimming test (FST) were performed in Wfs1-deficient mice. Simultaneously, gene expression and monoamine metabolism studies were conducted to evaluate changes in 5-HT- and NA-ergic systems of Wfs1-deficient mice. The basal immobility time of Wfs1-deficient mice in TST and FST did not differ from that of their wild-type littermates. However, a significant reduction of immobility time in response to lower doses of imipramine and paroxetine was observed in homozygous Wfs1-deficient mice, but not in their wild-type littermates. In gene expression studies, the levels of 5-HT transporter (SERT) were significantly reduced in the pons of homozygous animals. Monoamine metabolism was assayed separately in the dorsal and ventral striatum of naive mice and mice exposed for 30 min to brightly lit motility boxes. We found that this aversive challenge caused a significant increase in the levels of 5-HT and 5-hydroxyindoleacetic acid (5-HIAA), a metabolite of 5-HT, in the ventral and dorsal striatum of wild-type mice, but not in their homozygous littermates. Taken together, the blunted 5-HT metabolism and reduced levels of SERT are a likely reason for the elevated sensitivity of these mice to the action of imipramine and paroxetine. These changes in the pharmacological and neurochemical phenotype of Wfs1-deficient mice may help to explain the increased susceptibility of Wolfram syndrome patients to depressive states.

## Introduction

Wolfram syndrome (WS, MIM222300), caused by mutations in the WFS1 gene, is an autosomal recessive disorder most frequently characterized by diabetes insipidus, diabetes mellitus, optic atrophy, and deafness. In addition, around 60% of WS patients suffer from psychiatric disturbances related to depression, psychosis, impulsivity, and aggression (Swift et al., 1990). Heterozygous carriers of WFS1 mutations, not affected with WS, have a 26-fold higher likelihood of psychiatric hospitalization mainly due to depression (Swift and Swift, 2000). Therefore, it has been suggested that mutations in the WFS1 gene in humans play a marked role in the susceptibility to mood disorders (Swift et al., 1998; Koido et al., 2005; Swift and Swift, 2005).

We generated a Wfs1-deficient mouse line in order to study the possible role of Wfs1 protein in neuropsychiatric diseases. It has become evident that Wfs1-deficient mice exhibit more passive coping style in terms of increased avoidance behavior and higher plasma corticosterone concentration in stressful circumstances (Luuk et al., 2009). In humans, the prevalence of passive coping style (e.g., distancing, escape/avoidance behavior, high hypothalamus–pituitary–adrenal axis responsiveness) during stressful periods is associated with the development of stress-induced depression (Billings and Moos, 1984; Folkman and Lazarus, 1988; Patterson et al., 1993; Razzoli et al., 2007). Besides that, our recent data indicate a decreased activity of the dopaminergic (DA) system in Wfs1-deficient mice (Matto et al., 2011; Visnapuu et al., 2013). Impaired functioning of the DA-ergic system is likely connected to the development of mood disorders (Salamone et al., 2012). The prevalence of passive coping strategies and altered DA-ergic system possibly suggest that Wfs1-deficient mice could be an animal model of depressive states. Because of the apparently important role of serotonin (5-HT) and noradrenaline (NA) in the treatment of depression (Nemeroff and Owens, 2009; Goddard et al., 2010), we sought to study these neurotransmitter systems in Wfs1-deficient mice using pharmacological, genetic and biochemical methods.

Considering the efficacy of antidepressant drugs in the treatment of depression, we studied the effects of paroxetine (a selective 5-HT reuptake inhibitor) and imipramine (a 5-HT and NA reuptake inhibitor) in the tail suspension test (TST) and forced swimming test (FST) in Wfs1-deficient mice. Although both tests have often been used to describe the phenotype of genetically modified mice, they are not animal models of depression. Instead, these tests are simple and rapid animal models of behavioral despair used to screen the antidepressant-like effect of the drugs after their acute administration (Gardier, 2009). Since paroxetine selectively inhibits the 5-HT transporter (SERT) and imipramine mediates its effects through the inhibition of SERT and NA transporter (NAT), the gene expression levels of these transporters were measured in the mesencephalon and pons of naïve animals by using quantitative real-time PCR (qRT-PCR) analysis. The mesencephalon and pons were chosen because these brain structures are major seats of monoaminergic neurons, sending axon terminals to the forebrain structures, and playing a role in the development of depression (Malison et al., 1998; Drevets et al., 1999; Zhu et al., 1999).

Additionally, 5-HT and NA metabolism in the ventral and dorsal striatum was assessed by high performance liquid chromatography (HPLC) in response to an acute challenge of mice to brightly lit motility boxes. The ventral (involving nucleus accumbens and tuberculum olfactorium) and dorsal striatum (involving nucleus caudatus and putamen) were chosen because these brain structures regulate both motivations and emotions and, therefore, their impaired functioning is implicated in the mechanisms of depression (Nestler and Carlezon, 2006; Balleine et al., 2007; Krishnan and Nestler, 2008; Carlezon and Thomas, 2009).

## Materials and methods

### Animals

Wfs1-deficient mice were generated by invalidating the 8th exon of the Wfs1 gene (for details, see Luuk et al., 2008). Experiments were performed in 3–4 months old male and female F2 hybrids [(129S6/SvEvTac × C57BL/6) × (129S6/SvEvTac × C57BL/6)]. Two separate batches of naïve animals were used for the FST and TST. For the gene expression measurements, naïve animals, taken directly from their home-cages, were used. For the monoamine measurement studies, both naïve mice and mice exposed to motility boxes were chosen. Breeding and genotyping were conducted in the Department of Physiology, University of Tartu. The animals were kept in groups of eight per cage at 22 ± 1°C in a room illuminated artificially from 7 am to 7 pm. Tap water and food pellets were freely available. The permission (No. 88, 25th of August, 2011) for the present study was given by the Estonian National Board of Animal Experiments in accordance with the European Communities Directive of 24 November 1986 (86/609/EEC). Behavioral experiments were carried out between 10:00 and 17:00. Wfs1-deficient homozygous mice were always used in parallel with their wild-type and heterozygous littermates and the animals were randomly divided into experimental groups.

### Drugs

Control group animals in the TST and FST received an injection of saline (0.9% NaCl solution) (B. Braun Melsungen AG, Germany). Imipramine hydrochloride and paroxetine hydrochloride hemihydrate (both purchased from Sigma-Aldrich, St Louis, MO, USA) were dissolved in saline. Imipramine was administered at doses of 10, 20 and 30 mg/kg and paroxetine at doses of 5, 10, 20, and 30 mg/kg. All drugs were injected at a volume of 100 μl/10 g 40 min before using the animal in the TST or FST. Effect of paroxetine was studied only in TST since this test was more sensitive for establishing the antidepressant-like effect of drugs compared to FST (Liu and Gershenfeld, 2001).

### Behavioral studies

#### Tail suspension test

The TST has been extensively validated with a wide range of antidepressants (Porsolt et al., 1987). Most of the antidepressants maximally reduce the duration of immobility in the TST with doses less than those required for the FST (Liu and Gershenfeld, 2001). This test has been used alongside FST because hyperactivity may be a confounding issue in the FST. Mice were suspended from the edge of a shelf 58 cm above a tabletop by adhesive tape, placed approximately 1 cm from the tip of the tail. Animals were allowed to hang for 6 min and the duration of immobility was scored during the last 4 min from videotapes by an observer blind to the treatment protocol. Mice were considered immobile only when they hung passively and completely motionless.

#### Forced swimming test

The FST was performed as described by Porsolt et al. (1977). Briefly, a glass cylinder 12 cm in diameter was filled with 18 cm water at 25°C. The animal was gently put in the water, and the behavior recorded during 6 min. Subsequently, the immobility time was counted for the last 4 min of the test by an observer blind to the treatment protocol.

#### Motor activity test

Locomotor activity of mice was automatically registered for 30 min in photoelectric plexiglas motility boxes (448 × 448 × 450 mm, Technical and Scientific Equipment GmBH, Germany). The distance travelled, time in locomotion and number of corner entries were registered. Illumination level in the motility boxes during the experiments was approximately 400 lux, which is aversive and anxiogenic to rodents (Sütt et al., 2010). The floor of the motility boxes was cleaned thoroughly with 5% alcohol and dried after each animal.

### Gene expression studies

In the gene expression studies, experimentally naïve mice of all three genotypes were used in parallel. Mice were decapitated immediately after taking them out from their home-cage and bringing them into the room where the decapitation took place. The mesencephalon and pons were dissected according to the coordinates provided in the mouse brain atlas by Franklin and Paxinos (1997) and quickly frozen in liquid nitrogen.

#### RNA isolation, cDNA synthesis, and quantitative real-time-PCR

Total RNA was extracted individually from the mesencephalon and pons of each mouse using Trizol® Reagent (Invitrogen, USA) according to the manufacturer's protocol. RNA quality control was performed by Nanodrop where the ratios 260/230 and 260/280 were always around 2.00. For the first strand cDNA synthesis, 1 ug of total RNA of each sample was used with random hexamers (Applied Biosystems) and SuperScript™ III Reverse Transcriptase (Invitrogen, USA). For thermal cycling, a 7900HT Fast Real-time PCR system (Applied Biosystems) was used at 95°C for 10 min, then 40 cycles at 95°C for 15 s, and 60°C for 1 min. Assays for SERT and NAT (Applied Biosystems) and probes (Table 1) used in this study were designed from exon-exon junction eliminating the possibility of contamination with genomic DNA. HPRT1 was chosen for housekeeper gene, because our previous pairwise comparison experiments showed that HPRT is the most stably expressed reference gene in all genotypes as compared to GAPDH and β 2-microglobulin which are considered the other most commonly used housekeeping genes expressed in brain tissue (Raud et al., 2009). The PCR was performed in four parallel reactions for each sample. All experiments were repeated two times and negative control without template was always used on the plate. The amplification curve was similar in the case of housekeeper, SERT, and NAT genes. The amount for each transcript was calculated by a standard curve of cycle thresholds for serial dilutions of the complementary DNA sample and normalized to HPRT expression.

**Table 1:** **The taqman assays and probes used in the study**.

**Gene symbol**	**Assay ID or sequence**	**Gene ID**
Slc6a2	Mm00436661_m1	NM_009209.3
Slc6a4	Mm00439391_m1	NM_010484.2
Hprt1 for	5′-GCAGTACAGCCCCAAAATGG-3′	
Hprt1 rev	5′-AACAAAGTCTGGCCTGTATCCAA-3′	NM_013556
Hprt1 probe (VIC_TAMRA)	5′-VIC-AAGCTTGCTGGTGAAAAGGACCTCTCG TAMRA-3′	

Slc6a2, solute carrier family 6 (neurotransmitter transporter, NA), member 2; Slc6a4, solute carrier family 6 (neurotransmitter transporter, 5-HT), member 4; Hprt1, hypoxanthine phosphoribosyltransferase 1 gene.

### Monoamine measurements

Monoamines were measured in two groups: mice exposed for 30 min to the brightly lit motility boxes (exposure group) and mice taken directly from their home-cages (naïve group). The animals were immediately decapitated after completing the experiment in the motility boxes or after taking them out from their home-cage. Again, the animals were transported to a separate room for the decapitation. The dorsal (encompassing the nucleus caudatus and putamen) striatum and the ventral (encompassing the nucleus accumbens and tuberculum olfactorium) striatum were dissected according to coordinates by Franklin and Paxinos (1997). The dissected tissues were promptly frozen in liquid nitrogen. The tissue samples were homogenized with Bandelin Sonopuls ultrasonic homogenizer (Bandelin Electronic, Berlin, Germany) in ice-cold solution of 0.1 M perchloric acid (10–30 μ l/mg) containing 5 mM sodium bisulphite and 0.4 mM EDTA to avoid oxidation. The homogenate was then centrifuged at 17,000 × g for 10 min at 4°C. Aliquots (10 μ l) of the obtained supernatant were chromatographed on a Lichrospher 60 RP Select B column (250 × 3 mm; 5 μm). The separation was done in isocratic elution mode at column temperature of 30°C using the mobile phase containing 0.05 M sodium citrate buffer at pH 3.7; 0.02 mM EDTA; 1 mM KCl; 1 mM sodium octylsulphonate and 5.6% acetonitrile. The chromatography system consisted of a Hewlett Packard HP 1100 Series isocratic pump, a thermostatted autosampler, a thermostatted column compartment and an HP 1049 electrochemical detector (Agilent, Waldbronn, Germany) with glassy carbon electrode. The measurements were done at an electrode potential of + 0.7 V vs. the Ag/AgCl reference electrode. Tissue levels of 5-HT and NA (in pmol/mg) were determined using HPLC with electrochemical detection. Additionally, their respective metabolites 5-hydroxyindoleacetic acid (5-HIAA) and normetanephrine (NMN) were assayed.

### Statistics

The results of the behavioral and gene expression studies are expressed as mean values ± SEM. Since there were no significant sex differences in the results of any of the performed experiments, and to raise the statistical power of the study, data from male and female animals were pooled.

The results of the TST and FST were analysed using Two-Way ANOVA (genotype × treatment). One-Way ANOVA was applied for the statistical analysis of gene expression data. The results of monoamine and their metabolite assays were analysed using Two-Way ANOVA (genotype × exposure). *Post-hoc* comparisons were performed using Scheffe or Tukey HSD tests.

## Results

### Tail suspension test

#### Effects of imipramine and paroxetine

**Figure 1:**
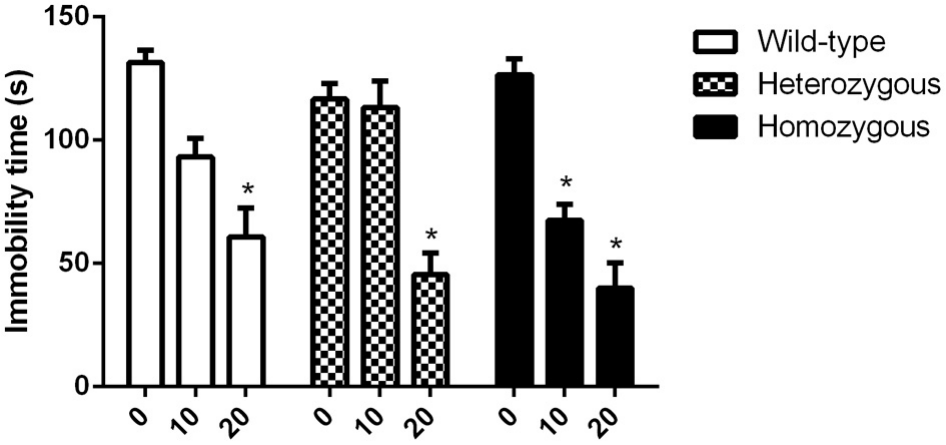
**Effect of imipramine on the immobility time of Wfs1-deficient mice in the TST**. ^*^p < 0.01 compared to vehicle-treated mice of the same genotype (Scheffe *post-hoc* test after significant Two-Way ANOVA). Altogether, 70 wild-type, 67 heterozygous and 71 homozygous mice were used. Mice were randomly divided between respective study groups. Genotype [*F*_(2, 199)_ = 4.2, *p* < 0.05]; treatment [*F*_(2, 199)_ = 84.1, *p* < 0.01]; genotype × treatment [*F*_(4, 199)_ = 3.5, *p* < 0.01].

There was no difference in the basal immobility levels between genotypes. It was found that in homozygous Wfs1-deficient mice, imipramine induced a significant decrease in immobility time at doses of 10 and 20 mg/kg as compared to vehicle treated homozygous mice (Figure 1). In both heterozygous and wild-type animals, only 20 mg/kg of imipramine was effective at significantly lowering immobility time compared to the respective vehicle-treated group of the same genotype.

**Figure 2:**
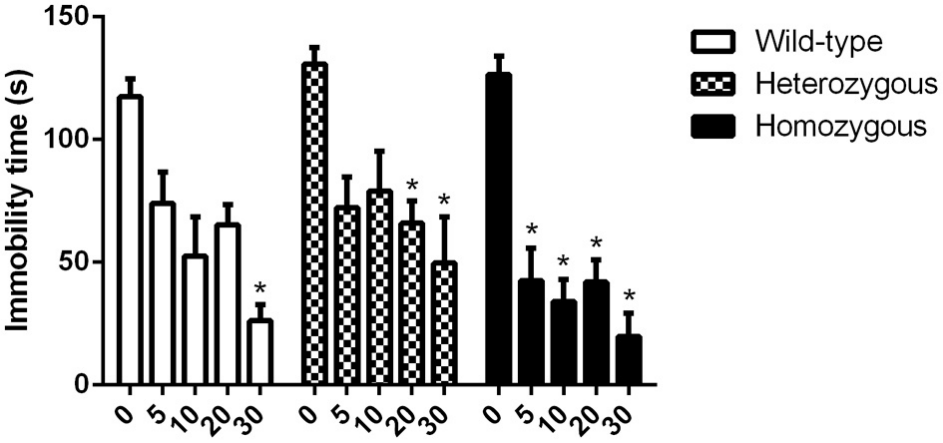
**Effect of paroxetine on the immobility time of Wfs1-deficient mice in the TST**. ^*^p < 0.01 compared to vehicle-treated mice of the same genotype (Scheffe *post-hoc* test after significant Two-Way ANOVA). Altogether, 93 wild-type, 95 heterozygous and 91 homozygous mice were used. Mice were randomly divided between respective study groups. Genotype [*F*_(2, 264)_ = 7.1, *p* < 0.01]; treatment [*F*_(4, 264)_ = 41.4, *p* < 0.01]; genotype × treatment [*F*_(8, 264)_ = 1.02, *p* = 0.42].

In the study where the effect of paroxetine was investigated, the basal immobility levels of all genotypes showed no statistically significant difference. In homozygous Wfs1-deficient mice, paroxetine induced a significant decrease in immobility time already at a dose of 5 mg/kg compared to vehicle group from the same genotype (Figure 2). For heterozygous animals, a significant difference in immobility time between vehicle-treated and drug-treated mice was established at doses of 20 and 30 mg/kg of paroxetine. In wild-type mice, only the highest dose (30 mg/kg) led to a significant reduction in immobility time compared to vehicle group from the same genotype (Figure 2).

### Forced swimming test

#### Effect of imipramine

**Figure 3:**
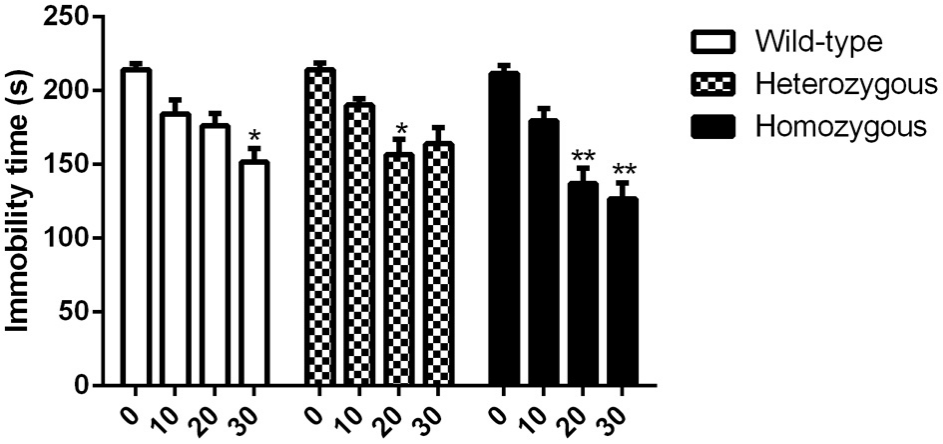
**Effect of imipramine on the immobility time of Wfs1-deficient mice in the FST**. ^*^p < 0.05, ^**^p < 0.01 compared to vehicle-treated mice of the same genotype (Scheffe *post-hoc* test after significant Two-Way ANOVA). Altogether, 77 wild-type, 80 heterozygous, and 79 homozygous mice were used. Mice were randomly divided between respective study groups. Genotype [*F*_(2, 224)_ = 5.9, *p* < 0.01]; treatment [*F*_(3, 212)_ = 37.1, *p* < 0.01]; genotype × treatment [*F*_(6, 224)_ = 1.7, *p* = 0.13].

In contrast to the TST, effective doses of imipramine were somewhat higher in this test. These results are in accordance with previous findings (Liu and Gershenfeld, 2001). As in the TST, basal immobility levels were similar across genotypes. For homozygous mice, a remarkable reduction in immobility behavior was observed at 20 and 30 mg/kg doses compared to vehicle-treated mice from the same genotype (Figure 3). Compared to vehicle received heterozygous mice, heterozygous animals treated with 20 mg/kg of imipramine showed significant decrease in immobility time whereas the highest dose (30 mg/kg) had no statistical effect. Finally, wild-type animals were sensitive only to the highest dose of imipramine as compared to drug-naïve wild-type mice (Figure 3).

### Gene expression studies

**Figure 4:**
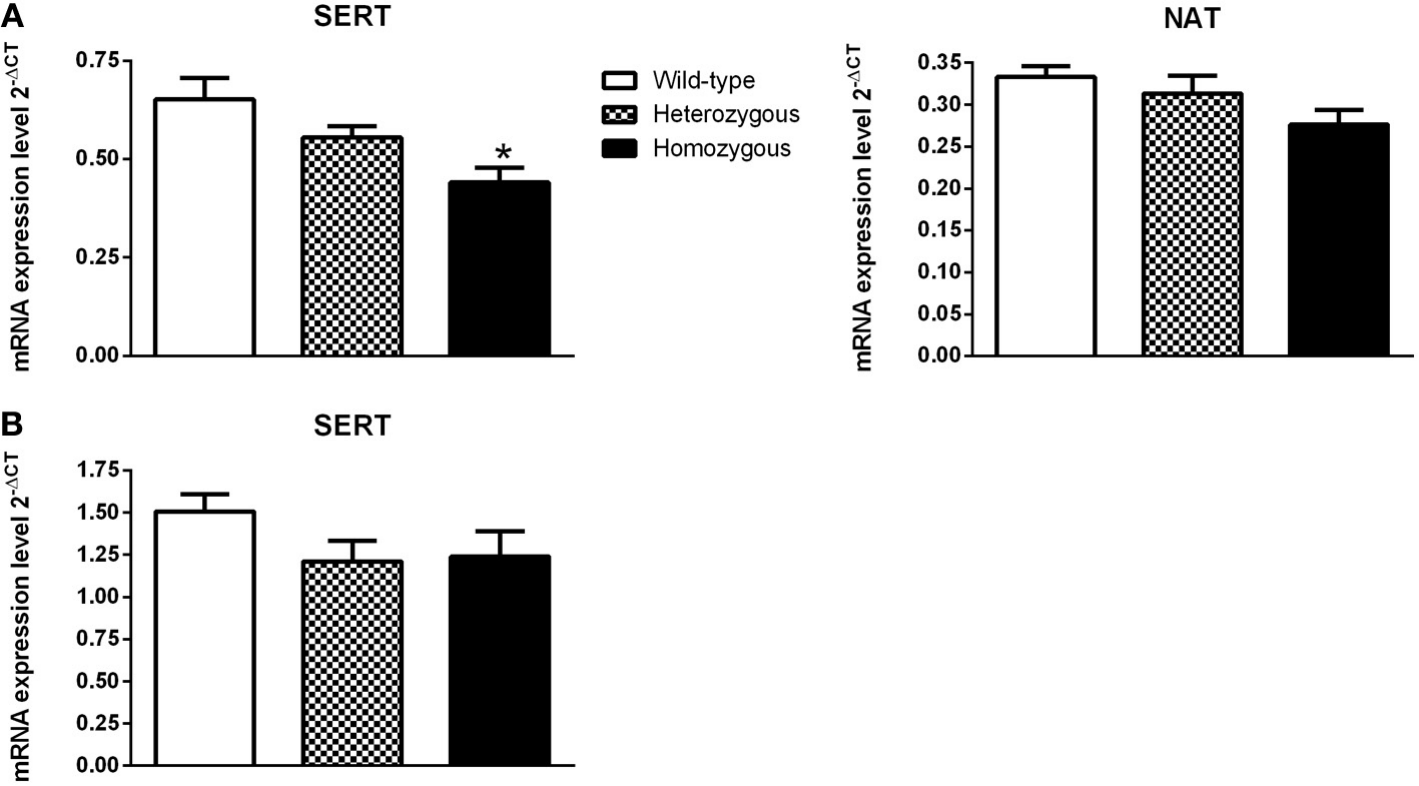
**Effect of Wfs1 gene invalidation on the expression of monoamine transporter genes in the pons (A) and mesencephalon (B)**. ^*^p < 0.01 compared to wild-type mice (Tukey HSD test after significant One-Way ANOVA). The number of mice in each group was 12–16. SERT (pons): genotype [*F*_(2, 41)_ = 6.49, *p* < 0.01]; NAT (pons): genotype [*F*_(2, 40)_ = 2.57, *p* = 0.09]. SERT (mesencephalon); genotype [*F*_(2, 39)_ = 1.76, *p* = 0.19].

In the pons, the expression of SERT was significantly lower in homozygous mice compared to their wild-type littermates (Figure 4A). The level of SERT mRNA in the mesencephalon was not changed in Wfs1-deficient mice (Figure 4B). The expression of NAT was lower in Wfs1-deficient mice in the pons compared to wild-type mice, but this difference did not reach statistical significance (Figure 4A). Furthermore, the levels of NAT mRNA in the mesencephalon were too low for any statistical analysis and, consequently, these data are not presented.

### 5-HT and NA measurements

There was no statistically significant difference in the locomotor activity of the three genotypes (data not shown). However, comparison between mice exposed to the motility boxes and experimentally naïve mice showed that exposure to the brightly lit motility boxes caused activation of the 5-HT-ergic, but not the NA-ergic, system in the dorsal and ventral striatum (Table 2). In the ventral striatum 5-HT and 5-HIAA levels were significantly increased in wild-type mice exposed to the motility boxes as compared to naïve wild-type littermates. The behavioral challenge failed to alter the levels of 5-HT and 5-HIAA in Wfs1-deficient homozygous mice, compared to naïve Wfs1-deficient homozygous mice (Table 2A). In heterozygous mice, this stressful challenge caused a rise in the levels of NA and 5-HIAA as compared to naïve heterozygous animals.

**Table 2:** **Effect of exposure of Wfs1-deficient mice to the motility boxes on the levels of monoamines and their metabolism in the ventral and dorsal striatum**.

	**Wild-type**	**Wild-type + exposure**	**Heterozygous**	**Heterozygous + exposure**	**Homozygous**	**Homozygous + exposure**
**(A) VENTRAL STRIATUM**						
NA	1.9 ± 0.3	2.4 ± 0.4	1.7 ± 0.2	3.7 ± 0.8^*^	2.7 ± 0.4	1.8 ± 0.3
NMN	1.0 ± 0.1	0.8 ± 0.1	0.8 ± 0.1	1.0 ± 0.1	0.8 ± 0.1	0.8 ± 0.1
5-HT	2.8 ± 0.2	3.9 ± 0.4^*^	3.2 ± 0.1	3.3 ± 0.2	3.2 ± 0.1	3.4 ± 0.2
5-HIAA	2.6 ± 0.2	3.9 ± 0.2^**^	2.7 ± 0.2	3.7 ± 0.2^**^	2.7 ± 0.1	3.3 ± 0.2
**(B) DORSAL STRIATUM**						
NA	2.4 ± 0.3	2.8 ± 0.6	2.2 ± 0.2	2.3 ± 0.2	3.1 ± 0.7	2.7 ± 0.2
NMN	0.7 ± 0.1	0.8 ± 0.1	0.8 ± 0.1	0.9 ± 0.1	0.8 ± 0.1	0.7 ± 0.1
5-HT	4.9 ± 0.1	6.1 ± 0.4	5.2 ± 0.1	5.8 ± 0.3	6.0 ± 0.4	5.3 ± 0.2
5-HIAA	1.8 ± 0.1	2.8 ± 0.2^**^	2.0 ± 0.2	3.3 ± 0.2^**^	2.1 ± 0.2	2.8 ± 0.2

* p < 0.05,

** p < 0.01 compared to naïve mice from the same genotype (Tukey HSD test after significant Two-Way ANOVA). There were 8–10 mice in each group.

5-HT (ventral striatum), exposure effect [F_(1, 46)_ = 5.62, p < 0.05], 5-HIAA (ventral striatum), exposure effect [F_(1, 46)_ = 34.4, p < 0.01]; NA (ventral striatum), genotype × exposure [F_(2, 46)_ = 4.98, p < 0.01]; 5-HIAA (dorsal striatum), exposure effect [F_(1, 51)_ = 41.6, p < 0.01]; 5-HT (dorsal striatum), genotype × exposure effect [F_(2, 51)_ = 4.85, p < 0.05].

In the dorsal striatum, 5-HIAA levels were significantly increased in wild-type and heterozygous mice exposed to the motility boxes as compared to their respective naïve litteramates. For homozygous animals, no difference was established in the level of 5-HIAA between the experimental and control groups (Table 2B). In wild-type mice, the level of 5-HT was not significantly increased (*p* = 0.09), whereas in homozygous mice, the concentration of 5-HT was reduced. Consequently, a simultaneous rise in wild-type mice and a decline in homozygous animals was the reason for a significant genotype × exposure interaction in Two-Way ANOVA analysis of 5-HT levels.

## Discussion

We have previously found a reduction in the activity of the DA-ergic system in Wfs1-deficient mice (Matto et al., 2011; Visnapuu et al., 2013). The present study adds to this knowledge the alteration of 5-HT-ergic, but not NA-ergic system in Wfs1-deficient mice. Statistical comparison of basal immobility times did not show significant differences between the three genotypes in either of the two conventional behavioral despair paradigms, the TST and FST. This finding is in accordance with the study by Kato et al. (2008), although a different model of Wfs1-deficient mice was used. Namely, they had invalidated the second exon of the Wfs1 gene and these Wfs1-deficient mice did not show marked deviations in the basal activity levels in the TST and FST (Kato et al., 2008). The present study extends this knowledge by demonstrating that the doses of paroxetine and imipramine, necessary for significantly reducing immobility time compared to drug-naive animals of the same genotype, are lower in homozygous mice than in their wild-type littermates. It has to be underlined that we did not find any sex-dependent differences and, therefore, the data from male and female mice were pooled. According to the radioligand binding studies, paroxetine shows significantly higher potency for SERT than for NAT (binding affinity for SERT 0.29–0.73 nM and for NAT 22–81 nM) (Shank et al., 1988; Bolden-Watson and Richelson, 1993; Hyttel, 1993). By contrast, the potency of imipramine is higher for NAT than for SERT (binding affinity for NAT 12–14 nM and for SERT 32–41 nM) (Shank et al., 1988; Bolden-Watson and Richelson, 1993; Owens et al., 1997; Richelson, 2001). Moreover, in accordance with previous studies (David et al., 2003; Ripoll et al., 2003), the antidepressant-like effect of imipramine was stronger in the TST than in the FST. In the TST, imipramine caused a significant reduction of immobility in wild-type mice at a dose 20 mg/kg, whereas for FST the effective dose was 30 mg/kg. In homozygous mice the effective doses were 10 and 20 mg/kg of imipramine, respectively. In the case of paroxetine, for wild-type mice, the effective dose of paroxetine was 30 mg/kg, whereas for homozygous mice it was 5 mg/kg. The established effective doses of imipramine and paroxetine are apparently higher than described in previous studies for FST and TST. These drugs usually display their antidepressant-like activity in mice at doses lower than 10 mg/kg (e.g., Ripoll et al., 2003). This apparent discrepancy can be explained by the genetic background of the mice. Most often, Swiss and C57Bl/6 strains have been exploited for studies of behavioral despair (Bai et al., 2001; David et al., 2003; Bourin et al., 2005; Jacobson and Cryan, 2007). In Swiss mice, both paroxetine and imipramine reduce immobility at dose of 8 mg/kg. For C57Bl/6 mice, paroxetine has anti-immobility effect already at 0.5 mg/kg (Ripoll et al., 2003), whereas effective dose of imipramine starts from 5 mg/kg in TST (Bai et al., 2001). In the present study, we used mice from a randomly mixed 129Sv × C57Bl/6 background. Because this background is not pure C57Bl/6, the established higher effective doses of imipramine and paroxetine are probably due to the 129Sv strain background. Our preliminary study, where effect of imipramine on immobility behavior of 129Sv mice in TST was studied, confirms this suggestion since 129Sv showed significant decrease in immobility time at doses of 20 and 30 mg/kg. Taking into account that the effectiveness of paroxetine was somewhat higher than that of imipramine, one could suggest that SERT has a more prominent role in the elevated behavioral response of homozygous mice.

The above-mentioned statement was explored further by measuring gene expression levels of SERT and NAT in experimentally naïve mice. Homozygous mice exhibited significantly lower expression of SERT mRNA in the pons as compared to their wild-type littermates. Moreover, the expression of SERT in the mesencephalon and NAT in the pons tended to be lower, but these differences were not statistically significant. Based on these findings, it can be speculated that the activity of SERT, but not NAT, is lower in Wfs1-deficient mice which may contribute to altered response to imipramine and paroxetine in these mice. Further studies for measuring SERT protein expression need to be done to confirm changes to the level of SERT in Wfs1-deficient mice.

The following step was to evaluate the effect of stressful challenge on the metabolism of 5-HT and NA in the dorsal and ventral striatum of Wfs1-deficient mice. Exposure to a brightly lit environment induced major changes in the levels of 5-HIAA, a metabolite of 5-HT. By contrast, we did not find any changes in the levels of NMN, a metabolite of NA, demonstrating a more prevalent role of 5-HT in the stress coping mechanism of mice. A statistically significant elevation of 5-HIAA levels was established in the dorsal and ventral striatum of heterozygous and wild-type mice. However, in the homozygous mice, the elevation of 5-HIAA was blunted. In wild-type mice, the levels of 5-HT tended to be elevated due to the stress-challenge, but this increase was statistically significant only in the ventral striatum. Therefore, the present study probably reflects stronger activation of 5-HT system in wild-type mice compared to Wfs1-deficient mice. Moreover, this finding, together with the reduced expression of SERT, can be indicative of the compromised function of 5-HT system in animals lacking the Wfs1 gene. We have found similar changes for DA in the dorsal and ventral striatum of Wfs1-deficient mice. Namely, the exposure of mice to an open field environment significantly increased the levels of homovanillic acid, a major metabolite of DA, in wild-type and heterozygous mice, but not in their homozygous littermates (Visnapuu et al., 2013). Moreover, this finding was accompanied by a reduced expression of DA transporter in homozygous animals. The impaired function of DA-ergic system was established also by Matto et al. (2011) where depolarization-induced release of DA was blunted in homozygous mice. Therefore, it is apparent that the functioning of two major monoaminergic systems (DA and 5-HT) is disturbed in Wfs1-deficient mice.

In conclusion, the present study demonstrates that lower doses of both paroxetine and imipramine are needed to induce antidepressant-like effect in Wfs1-deficient mice than in wild-type mice. Additionally, the expression of the SERT gene is lower and the response of the 5-HT-ergic system to a stressful challenge significantly reduced in homozygous animals compared to their wild-type littermates. Monoamine measurement findings and, to a certain degree, gene expression results can be taken as indicators of impaired function of 5-HT system due to the invalidation of the Wfs1 gene. This may explain behavioral findings in Wfs1-deficient mice treated with antidepressants in the two behavioral despair tests. Although the precise mechanism has to be further clarified, a possible reason for this manifestation could be the altered Ca^2+^ signaling observed in Wfs1-deficient mice. For instance, Ishihara et al. (2004) have demonstrated a reduced Ca^2+^-dependent stimulus-secretion coupling for insulin in Wfs1-deficient mice. Similar mechanism was hypothesized for the alterations occurring in the DA-ergic system (Matto et al., 2011; Visnapuu et al., 2013). Accordingly, one could claim that altered Ca^2+^ signaling is a possible reason for the observed changes in the function of the 5-HT-ergic system in Wfs1-deficient mice and the reduced gene expression of SERT is likely a compensatory change to the inhibited 5-HT release. To a certain extent, these findings concerning the compromised function of DA- and 5-HT-ergic systems, obtained from the animal studies, can be extended to patients suffering from WS. Therefore, it is possible that similar changes may occur in humans due to the invalid function of the WFS1 gene and this may explain an increase in the susceptibility of these patients to depressive disorders.

### Conflict of interest statement

The authors declare that the research was conducted in the absence of any commercial or financial relationships that could be construed as a potential conflict of interest.
